# Evaluating Behavioral Health Surveillance Systems

**DOI:** 10.5888/pcd15.170459

**Published:** 2018-05-10

**Authors:** Alejandro Azofeifa, Donna F. Stroup, Rob Lyerla, Thomas Largo, Barbara A. Gabella, C. Kay Smith, Benedict I. Truman, Robert D. Brewer, Nancy D. Brener, For the Behavioral Health Surveillance Working Group

**Affiliations:** 1 Substance Abuse and Mental Health Services Administration, Rockville, Maryland; 2 Data for Solutions, Inc, Atlanta, Georgia; 3 Michigan Department of Health and Human Services, Lansing, Michigan; 4 Colorado Department of Public Health and Environment, Denver, Colorado; 5 Centers for Disease Control and Prevention, Atlanta, Georgia; 6 Behavioral Health Surveillance Working Group

## Abstract

In 2015, more than 27 million people in the United States reported that they currently used illicit drugs or misused prescription drugs, and more than 66 million reported binge drinking during the previous month. Data from public health surveillance systems on drug and alcohol abuse are crucial for developing and evaluating interventions to prevent and control such behavior. However, public health surveillance for behavioral health in the United States has been hindered by organizational issues and other factors. For example, existing guidelines for surveillance evaluation do not distinguish between data systems that characterize behavioral health problems and those that assess other public health problems (eg, infectious diseases). To address this gap in behavioral health surveillance, we present a revised framework for evaluating behavioral health surveillance systems. This system framework builds on published frameworks and incorporates additional attributes (informatics capabilities and population coverage) that we deemed necessary for evaluating behavioral health–related surveillance. This revised surveillance evaluation framework can support ongoing improvements to behavioral health surveillance systems and ensure their continued usefulness for detecting, preventing, and managing behavioral health problems.

## Introduction

In 2015, more than 27 million people in the United States reported that they currently used illicit drugs or misused prescription drugs, and more than 66 million reported binge drinking during the previous month (1). The annual cost to the US economy for drug use and misuse is estimated at $193 billion, and the annual cost for excessive alcohol use is estimated at $249 billion (1). Death rates from suicide, drug abuse, and chronic liver disease have increased steadily for 15 years while death rates from other causes have declined (2). Such behavioral health problems are amenable to prevention and intervention (3). Because behavioral health care (eg, substance abuse and mental health services) has traditionally been delivered separately from physical health care rather than together, the Surgeon General’s report calls for integrating the 2 types of health care (1).

Public health surveillance and monitoring is critical to comprehensive health care (4–6). However, surveillance for behavioral health has been hindered by organizational barriers, limitations of existing data sources, and issues related to stigma and confidentiality (7). To address this gap, the Council of State and Territorial Epidemiologists (CSTE) has led the development of indicators for behavioral health surveillance (Box) (8) and has piloted their application in several states. CSTE’s rationale for selection of indicators was based on evidence for the need for such indicators and the feasibility of using them (8), suggesting that a national surveillance system for behavioral health is now achievable.

**Box. Indicators Recommended by the Council of State and Territorial Epidemiologists Working Group on Surveillance Indicators for Substance Abuse and Mental Health**

**Alcohol**

1. Adult binge drinking prevalence
2. Youth binge drinking prevalence
3. Alcohol-related crash death rate
4. Liver disease and cirrhosis death rate
5. State excise taxes on alcohol (beer, wine, distilled spirits)

**Other Drugs**

6. Drug overdose mortality rate
7. Hospitalization rate associated with drugs with potential for abuse and dependence
8. Prescription opioid sales per capita
9. Illicit drug or alcohol dependence or abuse in the past year
10. Prevalence of use of selected prescription and illicit drugs

**Mental Health**

11. Suicide death rate
12. Hospital discharge rate for mental disorders
13. Emergency department visit rate for intentional self-harm
14. Prevalence of youth suicide attempts
15. Prevalence of past-year major depressive episodes
16. Prevalence of past-year any mental illness
17. Prevalence of past-year serious mental illness
18. Prevalence of frequent mental distress

**Adapted from:** Council of State and Territorial Epidemiologists (8).

Routine evaluation of public health surveillance is necessary to ensure that any surveillance system provides timely, useful data and that it justifies the resources required to conduct surveillance. Existing surveillance evaluation guidelines (9,10) reflect a long history of surveillance for infectious diseases (eg, influenza, tuberculosis, sexually transmitted infections). Such guidelines present challenges for behavioral health surveillance. To address these challenges, CSTE convened a behavioral health surveillance working group of public health scientists and federal and state surveillance epidemiologists with experience in behavioral health surveillance and epidemiology. These experts came from the Substance Abuse and Mental Health Services Administration (SAMHSA), the Centers for Disease Control and Prevention (CDC), local and state health departments, and other partner organizations. The working group was charged with revising the published guidelines for evaluating public health surveillance systems (9,10) and extending them to the evaluation of behavioral health surveillance systems.

To lay a foundation for revising recommendations for evaluating behavioral health surveillance, the working group articulated concepts, characteristics, and events that occur more commonly with behavioral health surveillance than with infectious disease surveillance. First, behavioral health surveillance attributes are related to data source or indicator type, and evaluation should be made in the context of the data collection’s original purpose. For example, using mortality data for drug overdose deaths means that timeliness assessment is determined by availability of death certificate data, which are often delayed because of the time needed for toxicology testing. Second, traditional public health concepts may need adjustment for behavioral health. The concept of outcomes of interest (case definition) in behavioral health surveillance must be broadened to include health-related problems, events, conditions, behaviors, thoughts (eg, suicide ideation), and policy changes (eg, alcohol pricing). Third, clinical course of disease becomes a conceptual model for behavioral health. For example, behavioral health conditions may appear between precedent symptoms, behaviors, conditions, or exposure duration (from unhealthy stress or subclinical conditions), before the final appearance or diagnosis of disease or condition (eg, serious mental illness or substance use disorders). Fourth, behavioral health surveillance attributes are interrelated. For example, literature regarding data quality commonly includes aspects of completeness, validity, accuracy, consistency, availability, and timeliness (11). Finally, a gold standard for assessing some attributes might not be readily available (eg, a standard for suicide ideation). In lieu of a gold standard, 4 broad alternative methods can be used: regression approaches (12,13), simulation (14), capture–recapture methods (15), and network scale-up methods (16). The working group made modifications or revisions to the existing attributes of public health surveillance system evaluation and added 2 attributes (population coverage and informatics capabilities).

The purpose of this article is to summarize key definitions of attributes and methods for evaluating behavioral health surveillance systems developed by the working group. In addition, we present a logic model that portrays behavioral surveillance system theory and plausible associations between inputs and expected short-term, midterm, and long-term outcomes (Figure).

**Figure:**
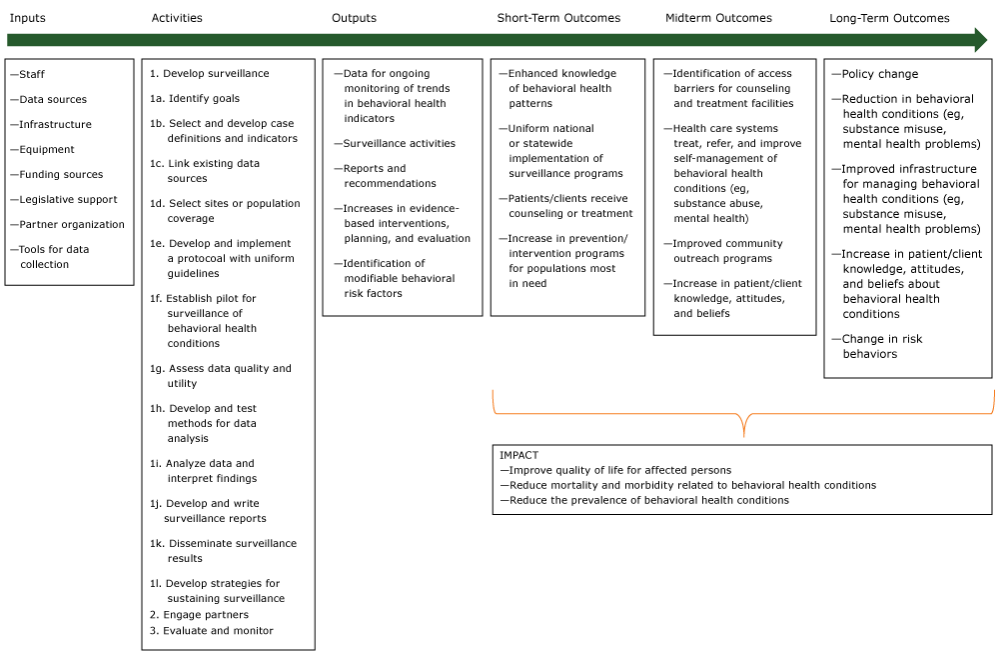
Logic model for behavioral health surveillance, adapted and used with permission from World Health Organization, Centers for Disease Control and Prevention, and International Clearinghouse for Birth Defects Surveillance and Research. Source: Birth defects surveillance: a manual for program managers. Geneva (CH): World Health Organization; 2014. http://apps.who.int/iris/bitstream/10665/110223/1/9789241548724_eng.pdf.

## Attributes for Evaluation of Behavioral Surveillance Systems

The working group provided definitions, recommended assessment methods, and reported on discussion of 12 behavioral surveillance system evaluation attributes the group recommended. Ten attributes are presented in order of existing evaluation guidelines (Table) (9) followed by the 2 new attributes.

**Table:** Existing Attributes for Evaluation of Public Health Surveillance Systems^a^

Attribute	Definition	Methods
Usefulness	A public health surveillance system is useful if it contributes to prevention and control of adverse health-related events, including an improved understanding of the public health implications of such events. A public health surveillance system can also be useful if it helps to determine that an adverse health-related event previously thought to be unimportant is actually important. In addition, data from a surveillance system can be useful in contributing to performance measures, including health indicators that are used in needs assessments and accountability systems.	An assessment of the usefulness of a public health surveillance system should begin with a review of the objectives of the system and should consider the system's effect on policy decisions and disease-control programs. Depending on the objectives of a particular surveillance system, the system might be considered useful if it satisfactorily addresses at least one of the following questions: Does the system detect diseases, injuries, or adverse or protective exposures of public importance in a timely way to permit accurate diagnosis or identification, prevention or treatment, and handling of contacts when appropriate? Does the system provide estimates of the magnitude of morbidity and mortality related to the health-related event under surveillance, including identification of factors associated with the event? Does the system detect trends that signal changes in the occurrence of disease, injury, or adverse or protective exposure, including detection of epidemics (or outbreaks)? Does the system permit assessment of the effect of prevention and control programs? Does the system lead to improved clinical, behavioral, social, policy, or environmental practices? Or Does the system stimulate research intended to lead to prevention or control? A survey of people who use data from the system might be helpful in gathering evidence regarding the usefulness of the system. The survey could be done either formally with standard methodology or informally.
Simplicity	Refers to the system’s structure and ease of operation. Systems should be as simple as possible.	Measures for determining simplicity include the amount and type of data necessary for establishing occurrence of the health-related event; amount and type of other data about cases; number of organizations involved in receiving case reports; integration with other systems; data collection, management, analysis, and dissemination procedures; amount of follow-up to update case data; staff training requirements; and time spent on maintaining the system.
Flexibility	Ability to adapt to changing information needs or technological operating conditions with little additional time, personnel, or allocated funds.	Probably best evaluated retrospectively by observing how a system has responded to new demands (eg, changes in case definitions, information technology, funding, or reporting sources).
Data quality	Refers to the completeness and validity of the data recorded in the system.	Measures for determining data quality include percentages of unknown, invalid, and missing responses to items on data-collection forms. In addition, data quality can be measured by applying edits for consistency in the data; however, a full assessment might require a special study.
Acceptability	Reflects the willingness of persons and organizations to participate in the system.	Measures for determining acceptability include subject or agency participation rate; interview completion rates and question refusal rates; completeness of reporting forms; physician, laboratory, or hospital or facility reporting rate; and timeliness of data reporting. A special study or survey might be required to obtain quantitative and qualitative data.
Sensitivity	Can be considered on at least 2 levels: at the level of case reporting, sensitivity refers to the proportion of cases of a disease (or event) detected by the system; on another level, it can refer to the ability to detect outbreaks over time. In evaluation of surveillance systems, completeness is often synonymous with sensitivity.	Assuming that reported cases are correctly classified, the primary emphasis in assessing sensitivity is on estimating the proportion of the total number of cases in the population under surveillance being detected by the system. The capacity for a system to detect outbreaks might be enhanced if detailed diagnostic tests are used. The measurement of sensitivity requires collection of or access to data usually external to the system to determine the true frequency of the condition and validation of data collected by the system. Also, the calculation of more than one measurement of the attribute might be necessary.
Predictive value positive (PVP)	The proportion of reported cases that actually are the event under surveillance.	Sensitivity and PVP provide different perspectives on how well the system is operating. Assessing PVP whenever sensitivity has been assessed might be necessary. In assessing this attribute, primary emphasis is placed on case confirmation, and records might be kept of investigations prompted by information obtained from the system. More than one PVP measurement might be necessary.
Representativeness	A public health surveillance system that is representative provides an unbiased indication over time and distribution of the extent of the problem measured by the surveillance system among the target population.	Representativeness is assessed by comparing the characteristics of the reported events to all such actual events. Although the latter information is generally not known, knowledge of the characteristics of the general population, clinical course of the disease or event, and prevailing medical practices, as well as collection of data from multiple sources, can be used to assess this attribute. Special studies based on samples of cases might be used. Also, the choice of an appropriate denominator for rate calculations should be given careful consideration.
Timeliness	Reflects the speed between steps in a system.	The time interval linking any of the steps in a system can be examined. These steps can include event occurrence, event recognition by reporting source, event reported to surveillance system, and control and prevention activities with feedback to stakeholders. The most relevant time interval might vary with the type of event under surveillance.
Stability	Refers to the system’s reliability (ability to collect, manage, and provide data without failure) and availability (ability to be operational when needed).	Measures for determining stability can include the number of unscheduled outages and down times for computer systems, the costs involved with any computer repair, the percentage of time the system is operating fully, and the desired and actual amount of time required for the system to collect, manage, and release data.

a Adapted from German et al (9).

### Usefulness

**Definition.** A public health surveillance system is useful if it contributes to preventing, treating, and controlling diseases, risk factors, and behaviors or if it contributes to implementation or evaluation of public health policies. Usefulness can include assessing the public health impact of a disease, risk, or behavior and assessing the status of effective prevention strategies and policies.

**Assessment methods.** Depending on its objectives, the surveillance system can be considered useful if it satisfactorily addresses one or more of the following questions:

- Does the system detect behavioral health outcomes, risk factors, or policies of public health importance, and does it support prevention, treatment, and control of these conditions?
- Does the system provide estimates of the magnitude of morbidity and mortality of the behavioral health conditions under surveillance?
- Does the system detect trends that signal changes in the occurrence of behavioral health conditions or clustering of cases in time or space?
- Does the system support evaluation of prevention, treatment, and control programs?
- Does the system “lead to improved clinical, behavioral, social, policy, or environmental practices” (9) for behavioral health problems?
- Does the system stimulate research to improve prevention, treatment, or control of behavioral health events under surveillance?

In addition to these attributes, a survey of people or stakeholders who use data from the system would be helpful in gathering evidence regarding the system’s usefulness.

**Discussion.** CSTE’s set of behavioral health indicators draws on 8 data sources: mortality data (death certificates), hospital discharge and emergency department data, the Behavioral Risk Factor Surveillance System (https://www.cdc.gov/brfss/index.html), the Youth Risk Behavior Surveillance System (https://www.cdc.gov/healthyyouth/data/yrbs/index.htm), prescription drug sales (opioids), state excise taxes for alcohol, the Fatality Analysis Reporting System (https://www.nhtsa.gov/research-data/fatality-analysis-reporting-system-fars), and the National Survey on Drug Use and Health (https://www.samhsa.gov/data/population-data-nsduh). These sources represent information regarding people, policies, and market data (eg, drug sales) and support different types of decisions for decision makers. Usefulness should be assessed in the context of the decision maker or interested stakeholders. In addition, surveillance data should provide clues to emerging problems and changing behaviors and products (eg, new drugs).

### Simplicity

**Definition.** A public health surveillance system is simple in structure and function if it has a small number of components with operations that are easily understood and maintained.

**Assessment methods.** Simplicity is evaluated by considering the system’s data-collection methods and the level to which it is integrated into other systems (9). For example, a surveillance system might rely on multiple information sources for case finding and data abstraction and for follow-up with confirmation by an independent data source or by an expert review panel. Evaluating simplicity would involve examining each data source individually and how the system works as a whole or how easily it integrates with other systems.

**Discussion.** As with infectious disease surveillance, behavioral health surveillance systems should be as simple as possible while still meeting the system’s objective and purpose. Each behavioral health indicator or outcome should have a clear definition and be measurable in that surveillance system. Surveillance systems using population survey methods should have simple standard sampling methods (eg, paper-based, computer-based, or telephone-based), data processing (eg, data cleaning, screening, weighting, and editing or imputing), and data dissemination (eg, reports, internet pages). Analysis of trends in behavioral health data assumes no change in variable definition(s) over time and that data elements are consistently defined when the numerator and denominator are taken from different data sources. This can entail defining or stabilizing a standard behavioral health case definition (eg, binge drinking differences between men and women) or diagnostic coding methods (eg, *International Statistical Classification of Diseases and Related Health Problems, 10th Revision* [17]). Simplicity is closely related to acceptance and timeliness (9) for detecting an event or outbreak.

### Flexibility

**Definition.** A system is flexible if its design and operation can be adjusted easily in response to a demand for new information. For example, the Behavioral Risk Factor Surveillance System (BRFSS) (https://www.cdc.gov/brfss/) allows flexibility for states to add questions (optional modules), adapting to new demands or to local health-related events or concerns, but it retains a core set of questions that allows state-to-state comparisons. The optional modules can address important state and nationwide emergent and local health concerns. The addition of new survey modules also allows the programs to monitor new or changing behaviors in the states. Moreover, states can stratify their BRFSS samples to estimate prevalence data for regions or counties within their respective states.

**Assessment methods.** Flexibility can be assessed retrospectively on the basis of historical evidence of response to change. A process map of steps needed to implement a change in the system as well as the following measures can address evaluation of flexibility:

- System technical design and change-process approval
- Time required to implement a change
- Number of stakeholders or organizations involved in agreement to implement a change (decision-making authority and system ownership, both important factors)
- Resources needed for change, including funding, technical expertise, time, and infrastructure
- Need for legacy (ie, continuity or legislative mandates) versus flexibility
- Time and process for validating and testing questions (eg, population-based surveys)
- Ability to add questions for specific stakeholders (eg, states, partner organizations) versus comparability for national estimates
- Ability to access subtopics
- Methods of data collection (eg, move from landlines to cellular telephones)
- Ability to deal with emerging challenges (eg, new or evolving recreational drugs)

**Discussion.** The Behavioral Health Surveillance Working Group recognizes different levels of flexibility. For example, BRFSS is flexible in terms of state-added questions, but adding a question to the core set is process-intensive. Flexibility should be assessed in the context of the data-collection purpose and the organization from which the data originate. For behavioral surveillance, flexibility to respond to changing norms and product availability is important.

### Data quality

**Definition.** System data quality is defined in terms of completeness and validity of data. Complete data have no missing values; valid data have no error (bias) caused by invalid codes or systematic deviation.

**Assessment methods.** For behavioral surveillance, measures of statistical stability (relative standard error) and precision (random variability and bias) are important. Completeness can be assessed at the item level (are values of a variable missing at random or clustering according to some characteristic?). Evaluation of completeness of the overall surveillance system can vary by data source. Completeness of a survey can be assessed by examining the sample frame (does it exclude groups of respondents?), sampling methodology, survey mode, imputation, weighting, and ranking methods (18). For behavioral surveillance based on medical records, consideration should be given to the completeness of all fields, standardization across reporting units (eg, medical records systems), coding process, and specific nomenclature (eg, for drugs and treatment). For surveillance based on death certificates, variability in death scene investigation procedures, presence of a medical examiner versus a coroner, reporting standards across geographic boundaries, and the process of death certification will be relevant.

Assessment of validity (ie, measurement of what is intended to be measured) also varies by data source. For use of data from a survey, consider cognitive testing of questions, focus groups, comparison with information from a health care provider, and identification of external factors that might influence reporting in a systematic way (19). An example of systematic influence is discrimination or prejudice in any form of arbitrary distinction, exclusion, or restriction affecting a person, usually (but not only) because of an inherent personal characteristic or perceived membership of a particular group (20).

Evaluation of statistical stability (precision) involves calculation of relative standard error of the primary estimate. Assessment of bias (systematic error) should address the following:

- Selection bias: systematic differences between sample and target populations
- Performance bias: systematic differences between groups in care provided or in exposure to factors other than the interventions of interest
- Detection bias: systematic differences in how the outcome is determined (eg, death scene investigation protocols)
- Attrition bias: systematic loss to follow up
- Reporting bias: systematic differences in how people report symptoms or ideation
- Other: biases related to a particular data source

**Discussion.** Many data-quality definitions depend on other system performance attributes (eg, timeliness, usefulness, acceptability) (21). Because of reliance on multiple data sources, data quality must be assessed in different ways. For surveillance relying on surveys, concepts of reliability, validity, and comparison with alternative data sources are important. For example, considerations of possible data-quality concerns arise with use of mortality data, particularly underreporting of suicide.

### Acceptability

**Definition.** Acceptability is the willingness of individuals and groups (eg, survey respondents, patients, health care providers, organizations) to participate in a public health surveillance system (9).

**Assessment methods.** For behavioral surveillance, acceptability includes the willingness of people outside the sponsoring agency to report accurate, consistent, complete, and timely data. Factors influencing the acceptability of a particular system include

- Perceived public health importance of a health condition or behavior, risk factor, thought, or policy
- Nature of societal norms regarding the risk behavior or outcome (discrimination or stigma)
- Collective perception of privacy protection and government trustworthiness
- Dissemination of public health data to reporting sources and interested parties
- Responsiveness of the sponsoring agency to recommendations or comments
- Costs to the person or agency reporting data, including simplicity, time required to enter data into the system, and whether the system is passive or active
- Federal and state statutes ensuring privacy and confidentiality of data reported
- Community participation in the system

When a new system imposes additional reporting requirements and increased burden on public health professionals, acceptability can be indicated by topic-specific or agency-specific participation rate, interview completion and question refusal rates, completeness of reporting, reporting rate, and reporting timeliness.

**Discussion**. Assessment of acceptability includes considerations of other attributes, including simplicity and timeliness. Acceptability is directly related to the extent to which the surveillance system successfully addresses stigma associated with certain conditions, which is particularly important for behavioral surveillance, in terms of both the extent to which the questions included in the survey questionnaire are sensitive to the reluctance people may have to report various behavioral health problems and the nonjudgmental quality of questions.

### Sensitivity

**Definition.** Sensitivity is the percentage of true behavioral health events, conditions, or behaviors occurring among the population detected by the surveillance system. A highly sensitive system might detect small changes in the number, incidence, or prevalence of events occurring in the population as well as historical trends in the occurrence of behavioral health events, conditions, or behaviors. Sensitivity may also refer to the ability to monitor changes in prevalence over time, including the ability to detect clusters in time, place, and segments of the population requiring investigation and intervention.

**Assessment methods.** Measurement of the sensitivity of a public health surveillance system is affected by the likelihood that

- Health-related events, risk factors, or effects of public health policies are occurring in the population under surveillance
- Cases are coming to the attention of institutions (eg, health care, educational, community-based, harm-reduction, law enforcement, or survey-collection institutions) that report to a centralized system
- Cases will be identified, reflecting the abilities of health care providers; capacity of health care systems; type, quality, or availability of the screening tool; or survey implementation
- Events will be reported to the system. For example, in assessing sensitivity of a surveillance system based on a telephone-based survey, one can assess the 1) likelihood that people have telephones to take the call and agree to participate; 2) ability of respondents to understand the questions and correctly identify their status and risk factors, and 3) willingness of respondents to report their status.

Because many important conditions for behavioral health surveillance are self-reported, validating or adjusting the self-report might be required using statistical methods (10), field-based studies (16), or methods in the absence of a gold standard (12–15).

Other factors related to behavioral health (eg, discrimination and variability in implementing parity in payment coverage between physical health and behavioral health care) can influence sensitivity, requiring alternative or parallel data sources. For example, when using surveys as a source for prevalence data, consider question redundancy or adding questions that might further identify people with a condition or leading indicator.

**Discussion.** An evaluation of the sensitivity of a behavioral health surveillance system should include a clear assessment of potential biases that range from case identification to case reporting. Case identification and case reporting will require workforce capacity, ability, and willingness to accurately and consistently identify and report plus an organized system for collecting, collating, and aggregating identified cases.

### Predictive value positive

**Definition.** Predictive value positive (PVP) is the proportion of reported cases that actually have the health-related event, condition, behavior, thought, or policy under surveillance.

**Assessment methods.** PVP’s effect on the use of public health resources has 2 levels: outbreak identification and case detection. First, PVP for outbreak detection is related to resources; if every reported case of suicide ideation is investigated and the community involved is given a thorough intervention, PVP can be high, but at a prohibitive expense. A surveillance system with low PVP (frequent false-positive case reports) might lead to misdirected resources. Thus, the proportion of epidemics identified by the surveillance system that are true epidemics can be used to assess PVP. Review of personnel activity reports, travel records, and telephone logbooks may be useful. Second, PVP might be calculated by analyzing the number of case investigations completed and the proportion of reported persons who actually had the behavioral health-related event. However, use of data external to the system (eg, medical records, registries, and death certificates) might be necessary for confirming cases as well as calculating more than one measurement of the attribute (eg, for the system’s data fields, for each data source or combination of data sources, for specific health-related events).

**Discussion.** Although the definition of PVP is the same as for infectious conditions, measuring PVP for behavioral health surveillance is hindered by a lack of easily measurable true positives as a result of stigma, communication, or cultural factors. Approaches cited previously for evaluating accuracy in absence of a gold standard can be helpful (12–16) in addition to the use of alternative data sources (eg, medical records, police reports, psychological autopsies), redundant questions within a survey (for survey-based surveillance), longitudinal studies, or follow-up studies.

### Representativeness

**Definition.** A behavioral health surveillance system is representative if characteristics of the individuals (or people) assessed by the system as essentially the same as the characteristics of the population subject to surveillance.

**Assessment methods.** Assessment of representativeness requires definition of the target population and of the population at risk, which can differ. Examination of groups systematically excluded by the surveillance data source (eg, prisoners, homeless or institutionalized persons, freestanding emergency departments, people aged ≥65 in Veterans Affairs systems) can help to assess representativeness. An independent source of data regarding the outcome of interest is also helpful. Using behavioral health event data requires calculation of rates for a given year or for monitoring temporal trends. These will use denominator data from external data sources (eg, US Census Bureau ) that should be carefully ascertained for the targeted population. These considerations facilitate representation of health events in terms of time, place, and person.

**Discussion.** Generalizing the findings of surveillance to the overall population should be possible with data captured from the surveillance system. Although sensitivity is the proportion of all health events of interest captured by the system, representativeness quantifies whether the data system accurately reflects the distribution of the condition or affected individuals in the general population (ie, whether systematic errors exist). For example, because many emergency departments and trauma centers that treat acute injuries test only a limited proportion of patients for alcohol, data regarding alcohol involvement in nonfatal injuries might not be representative of alcohol involvement in injuries overall. Generalization from these findings on alcohol involvement in nonfatal injuries to all persons who have experienced these outcomes is problematic. Alternative survey methods are useful — respondent-driven sampling (22), network scale-up methods (16), and time/date/location sampling (23). Evaluation of representativeness can prompt modification of data-collection methods or redefining and accessing the target population to accurately represent the population of interest.

### Timeliness

**Definition.** Timeliness reflects the rate at which the data move from occurrence of the health event to public health action.

**Assessment methods.** Evaluating timeliness of behavioral health systems will depend on the measure used (eg, symptom, event, condition) and the system’s purpose. Timeliness of a behavioral health surveillance system should be associated with timing of a consequent response for detecting a change in historical trends, outbreaks, or policy to control or prevent adverse health consequences. For example, quick identification and referral is needed for people experiencing a first episode of psychosis. However, for a community detecting an increase in binge-drinking rates, a longer period will be needed because the public health response requires a systemic engagement at the community level. Specific factors that can influence timeliness include

- Delays from symptom onset to diagnosis resulting from stigma (people might avoid diagnosis), lack of access to a facility or practitioner for diagnosis, policy (providers might be unable to bill for behavioral health diagnoses), credentials (relying on medical records or insurance claims misses people without insurance), or a failure to diagnose to avoid labeling
- Case definitions (eg, requiring symptoms be present for ≥6 months)
- A symptom that might be associated with multiple possible diagnoses, taking time to resolve
- Symptoms that appear intermittently
- Variance in detection methods
- Delays in recognizing a cluster or outbreak caused by lack of baseline data

**Discussion.** For behavioral health conditions, long periods can occur between precedent symptoms, behavior, conditions, or exposure duration and the final appearance or diagnosis of a disease or condition. Unlike immediate identification and reporting needed for infectious diseases, some behavioral health conditions, similar to chronic conditions, might develop more slowly; for example, posttraumatic stress disorder (which often occurs in response to a particular traumatic event over time) versus an episodic depression (which may occur in response to an acute event). Nonetheless, baseline data are vital for determining the urgency of timely response to outbreaks or clusters of health problems related to behavioral health conditions. Ultimately, timeliness should be guided by the fact that behavioral health measures are not as discrete or easily measureable as most chronic or infectious disease measures, and their etiology or disease progression is often not as linear.

### Stability

**Definition.** Stability of a public health surveillance system refers to a system’s reliability (ability to collect, manage, and provide data dependably) and availability (ability to be operational when needed).

**Assessment methods.** The system’s stability might be assessed by protocols or model procedures based on the purpose and objectives of the surveillance system (9). Changes in diagnostic criteria or in the availability of services can affect stability. When relying on surveys, check the stability of questions and survey design. Assessing the system’s workforce stability and continuity should include staff training, retention, and turnover. Existing measures for evaluating the stability of the surveillance system might be applicable for behavioral health surveillance systems (9).

**Discussion.** The stability of a behavioral health surveillance system will depend on the operational legal or regulatory framework on which the surveillance system is based. For example, an established legal or regulatory framework ensures continuity in system funding and workforce capacity. Stability should be maintained while allowing flexibility to adapt to emerging trends. Assessing the stability of a surveillance system should be based on the purpose and objectives for which the system was designed.

### Informatics capabilities

**Definition.** Public health informatics is the systematic application of information and computer science and technology to public health practice, research, and learning (24). Public health informatics has 3 dimensions of benefits to behavioral health surveillance: the study and description of complex systems (eg, models of behavioral health development and intervention), the identification of opportunities to improve efficiency and effectiveness of surveillance systems through innovative data collection or use of information, and the implementation and maintenance of surveillance processes and systems to achieve improvements (25).

**Assessment methods.** When assessing informatics components of a surveillance system, the following aspects should be considered (25):

- Planning and system design: identifying information and sources that best address a surveillance goal; identifying who will access information, by what methods, and under what conditions; and improving interaction with other information systems
- Data collection: identifying potential bias associated with different collection methods (eg, telephone use or cultural attitudes toward technology); identifying appropriate use of structured data, vocabulary, and data standards; and recommending technologies to support data entry
- Data management and collation: identifying ways to share data across computing or technology platforms, linking new data with legacy systems, and identifying and remedying data-quality problems while ensuring privacy and security
- Analysis: identifying appropriate statistical and visualization applications, generating algorithms to detect aberrations in behavioral health events, and leveraging high-performance computational resources for large data sets or complex analyses
- Interpretation: determining usefulness of comparing information from a surveillance program with other data sets (related by time, place, person, or condition)
- Dissemination: recommending appropriate displays and best methods for reaching the intended audience, facilitating information finding, and identifying benefits for data providers
- Application to public health programs: assessing the utility of having surveillance data directly support behavioral health interventions

**Discussion.** Initial guidelines for infectious disease surveillance (4) did not include assessment of informatics capability. Although this was included in a later publication (10), informatics was not portrayed as an attribute for evaluation. Because of the proliferation of electronic medical records and the standards for electronic reporting, assessment of informatics as an attribute will be crucial for behavioral health surveillance.

### Population coverage

**Definition.** Population coverage refers to the extent that the observed population described by the data under surveillance describes the true population of interest.

**Assessment methods.** Population coverage can be assessed by the proportion of respondents (survey-based) or cases (hospital- or facility-based) included in the surveillance system. Two measurements resulting from population coverage assessment are 1) population undercoverage that results from the omission of respondents or cases belonging to the target population and 2) population overcoverage that occurs because of inclusion of elements that do not belong to the target population. In addition, a demographic analysis (26) can provide benchmarks for assessing completeness of coverage in the existing surveillance data and document changes in coverage from previous periods. Furthermore, independence and internal consistency of the demographic analysis allow using estimates to check survey-based coverage estimates.

**Discussion.** Surveillance systems (ie, survey-based or hospital- or facility-based surveillance) can be defined by their geographic catchment area (ie, country, region, state, county, or city) or by the target population that the system is intended to capture. For example, the National Survey on Drug Use and Health’s target population is the noninstitutionalized civilian population aged 12 years or older. Homeless people who do not use shelters, active duty military personnel, and residents of institutional group quarters (eg, correctional facilities, nursing homes, mental institutions, and long-term hospitals) are excluded. Such populations not covered by most surveillance systems can contribute to case counts in hospital- or facility-based systems (eg, drug poisoning, emergency department use for self-harm, prevalence of mental illness and substance abuse problems). Evaluation of population coverage typically requires an alternative data source. For example, the estimate from a national surveillance system can be compared with a special study or survey in the same geographic area targeting a specific population. Projections from previous estimates might aid in comparing existing surveillance data. Use of benchmark data sets might aid in estimating the undercoverage prevalence of behavioral health indicators: the US Department of Justice’s Bureau of Justice Statistics data (https://www.bjs.gov/index.cfm?ty=dca) and the US Department of Housing of Urban Development’s (http://portal.hud.gov/hudportal/HUD) point-in-time estimates of homelessness. Finally, mortality data will contain all US residents’ deaths occurring in a given year; however, residents who die abroad might not be included (resulting in undercoverage), and deaths of nonresidents might be included (resulting in overcoverage).

## Conclusions and Recommendations

The increasing burden of behavioral health problems despite the existence of effective interventions argues that surveillance for behavioral health problems is an essential public health function. In establishing surveillance systems for behavioral health, guidelines for periodic evaluation of the surveillance system are needed to ensure continued usefulness for design, implementation, and evaluation of programs for preventing and managing behavioral health conditions. We developed the framework described in this article to facilitate the periodic assessment of these systems.

Recommendations for improving a behavioral health surveillance system should clearly address whether the system should continue to be used and whether it might need to be modified to improve usefulness. The recommendations should also consider the economic cost of making improvements to the system and how improving one attribute of the system (eg, population coverage) might affect another attribute, perhaps negatively (eg, simplicity). The results of a pilot implementation, in collaboration with stakeholders, should help determine whether the surveillance system is addressing an important public health problem and is meeting its objective of contributing to prevention and intervention for behavioral health problems.

This revised framework could be implemented in future evaluations of the behavioral health surveillance systems at any level. As behavioral health issues become more relevant and local authorities enhance or develop behavioral surveillance systems, this framework will be helpful for such evaluation. Finally, because behavioral health theories, survey technology, public health policies, clinical practices, and availability of substances continue to evolve, this framework will need to adapt.
